# Obtaining complete and canonical ammonia-oxidizing bacteria through specific labeling and cell sorting

**DOI:** 10.1093/ismeco/ycae145

**Published:** 2025-02-08

**Authors:** Pieter Blom, Pascal C Huizing, João P R C de Monlevad, Maartje A H J van Kessel, Sebastian Lücker

**Affiliations:** Department of Microbiology, Radboud Institute for Biological and Environmental Sciences, Radboud University, Nijmegen, Heyendaalseweg 135, 6525 AJ, Nijmegen, The Netherlands; Department of Microbiology, Radboud Institute for Biological and Environmental Sciences, Radboud University, Nijmegen, Heyendaalseweg 135, 6525 AJ, Nijmegen, The Netherlands; Department of Microbiology, Radboud Institute for Biological and Environmental Sciences, Radboud University, Nijmegen, Heyendaalseweg 135, 6525 AJ, Nijmegen, The Netherlands; Department of Microbiology, Radboud Institute for Biological and Environmental Sciences, Radboud University, Nijmegen, Heyendaalseweg 135, 6525 AJ, Nijmegen, The Netherlands; Department of Microbiology, Radboud Institute for Biological and Environmental Sciences, Radboud University, Nijmegen, Heyendaalseweg 135, 6525 AJ, Nijmegen, The Netherlands

**Keywords:** targeted isolation, ammonia-oxidizing bacteria, fluorescence-activated cell sorting, cultivation, Nitrosomonas, Nitrospira, comammox bacteria

## Abstract

Mitigation of the negative environmental consequences of excess anthropogenic nitrogen input requires a thorough understanding of the processes driving the biogeochemical nitrogen cycle. Nitrification is one of the key nitrogen-cycling processes and is performed by ammonia-oxidizing bacteria and archaea, nitrite-oxidizing bacteria, and complete nitrifiers. However, the fastidious growth of nitrifiers largely hampered their isolation using classical cultivation techniques, as most nitrifiers do not grow on solid media. Here, we present a workflow for the targeted enrichment and isolation of complete and canonical ammonia-oxidizing bacteria by combining function-specific in vivo fluorescent labeling with cell sorting. Optimized floc disruption and labeling techniques enlarged the fraction of planktonic cells and the fluorescent signal intensity, respectively, while maintaining cell viability. Sorted fractions were incubated in ammonium-containing mineral media and were screened for nitrite and nitrate production. Nitrifying cultures were upscaled and characterized with 16S ribosomal ribonucleic acid and *amoA* gene-targeted polymerase chain reactions and fluorescence *in situ* hybridization. Overall, we obtained one axenic and one enriched *Nitrosomonas*, and seven comammox *Nitrospira* enrichment cultures from five bioreactors, a recirculating aquaculture system biofilter, and agricultural soil. In conclusion, the presented workflow enables the fast and targeted retrieval of ammonia oxidizers from complex samples, allowing for in-depth physiological characterization.

## Introduction

The biogeochemical nitrogen cycle is beyond the safe planetary boundaries and currently operates in the high-risk zone [1, 2]. These risks include increased nitrogen flow into terrestrial and aquatic ecosystems, resulting in eutrophication [3] and, consequently, a reduction in water quality and the formation of anoxic zones [4]. Increased nitrogen concentrations also result in elevated production of nitrous oxide, a strong greenhouse gas contributing to global warming [5]. Furthermore, higher emissions of nitrogen oxides negatively affect air quality, especially in urbanized regions, with severe effects on human health [6, 7].

Nitrification, the microbial conversion of bioavailable ammonia via nitrite to nitrate, is one of the key processes of the biogeochemical nitrogen cycle. This process can be both harmful and beneficial from a societal perspective. In agricultural settings, nitrification results in the loss of bioavailable nitrogen as ammonium adsorbs well to soil particles and can readily be taken up by plant roots, whereas nitrate leaches from soil into attached water bodies and the groundwater [8]. In drinking water (DWTPs) and wastewater treatment plants (WWTPs), however, nitrification drives the removal of nitrogen from the incoming water. This nitrogen removal ultimately prevents biological growth in drinking water distribution systems, making the water safe for human consumption [9], and decreases the risk of eutrophication in water bodies receiving WWTP effluents [10].

It was a long-standing paradigm of the nitrogen cycle that nitrification is a two-step process performed by distinct guilds of ammonia-oxidizing archaea (AOA) and bacteria (AOB) performing the conversion of ammonia to nitrite, which subsequently is converted to nitrate by nitrite-oxidizing bacteria (NOB). Due to the availability of multiple cultured representatives, the physiological properties of most known taxonomic groups of AOA and AOB have been relatively well characterized. AOB were initially considered to be more copiotrophic and have lower affinities for ammonia than AOA [11], but in soil ecosystems, they generally coexist. Recent studies reveal a more nuanced view where certain clusters of AOB can compete with terrestrial lineages of AOA in terms of ammonia affinity [12, 13].

The discovery of bacteria performing complete ammonia oxidation (comammox) overturned this two-step paradigm by showing that members of the genus *Nitrospira* have the genetic and physiological potential to perform both steps of nitrification [14, 15]. Based on the theory of optimal pathway length, comammox bacteria were predicted to have high substrate affinities, high biomass yields, and low growth rates [16]. Physiological data on the few available comammox species support these predictions and show that they have high ammonia affinities that fall between those of terrestrial and marine AOA [17]. However, an unexpectedly low nitrite affinity was reported for *Nitrospira inopinata* [17] but not for Ca. Nitrospira kreftii [18]. Conversely, Ca. N. kreftii showed inhibition at ammonium concentrations >25 μM [18], which was not observed for *N. inopinata*.

Based on *amoA* sequence phylogeny, comammox *Nitrospira* can be divided into two sister groups referred to as clades A and B. However, all cultures available to date belong to comammox clade A and our understanding of the ecophysiology of members of clade B is limited. Additionally, the physiological data collected for phylogenetically diverse AOA and AOB illustrate that new isolates can provide a more nuanced understanding of their physiology. Consequently, the discrepancies between the physiological data of comammox cultures can only be resolved by the availability of new isolates, but most nitrifiers show poor cultivability due to their slow and fastidious growth. Nitrifying cultures have been obtained by classical cultivation methods such as physical separation [19, 20] and continued serial dilution-to-extinction [17, 21]. However, physical separation techniques using solidifying agents fail to capture the diversity of several groups of nitrifiers such as *Nitrospira,* which have not yet been demonstrated to form colonies on solid media. Moreover, continued serial dilution-to-extinction has the propensity to select for the fastest-growing nitrifiers, which are the species adapted to higher substrate concentrations.

Fluorescence-activated cell sorting poses an interesting alternative to these challenges as it allows the physical separation of single cells or clonal cell clusters without the use of solidifying agents. Large-scale untargeted cell sorting approaches have yielded many new isolates from the human gut microbiome [22, 23], and also axenic nitrifying cultures of *Nitrospira* and *Nitrosomonas* have been obtained by untargeted sorting [24, 25]. The use of targeted labeling and sorting approaches such as bioorthogonal non-canonical amino acid tagging (BONCAT) allowed the retrieval of the active fraction of microbial communities [26, 27]. Another targeted approach, which fluorescently labels all copper membrane monooxygenase (CuMMO)-containing microorganisms using activity-based protein profiling (ABPP), has already been successfully applied on fixed biomass for targeted metagenomics [28] and might be applicable to living biomass to selectively isolate (complete) ammonia oxidizers.

**Figure 1:**
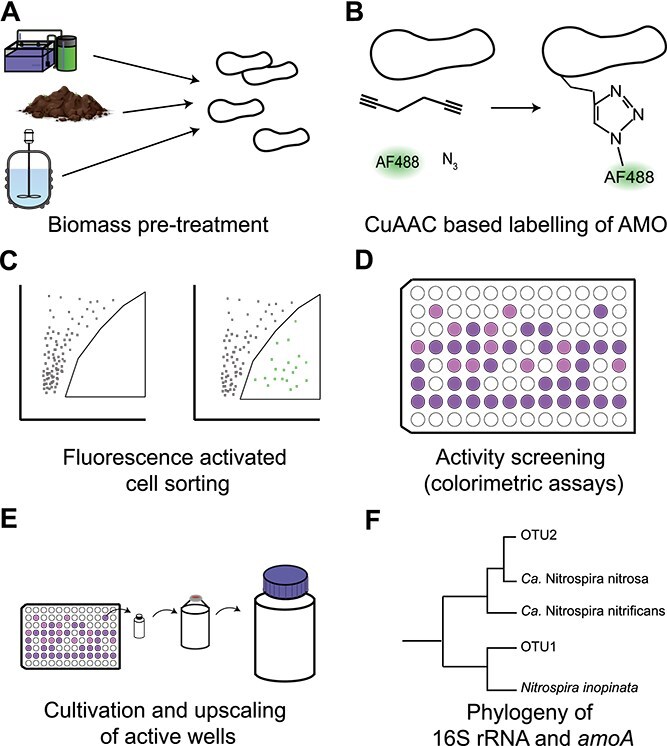
Experimental workflow for the targeted enrichment and isolation of ammonia oxidizers. (A) Biomass samples are pretreated to obtain small flocs and planktonic cells. (B) CuMMO-containing cells are fluorescently labeled using the CLICK reaction. (C) Fluorescently-labeled cells are subsequently sorted into culture plates using FACS. (D) Sorted cells are incubated and tested for nitrite and/or nitrate formation. (E) After observing nitrite or nitrate formation, wells containing active cells will be transferred to bigger volumes. (F) The obtained cultures can be characterized.

In this study, a workflow is designed to obtain new ammonia-oxidizing cultures from both natural and engineered systems by combining this specific CuMMO labeling with live cell sorting (Fig. 1). In order to optimize cell viability for downstream cultivation, the performance of several flock disruption methods, fluorescent labeling strategies, and cultivation media was compared. The nitrifying activity of sorted fractions was screened, and active cultures were upscaled. Finally, active nitrifying cultures were taxonomically classified and assessed for their level of enrichment.

## Materials and methods

### Biomass collection and extraction

Biomass (1.5–50 mL) from the following bioreactors was collected: both compartments of a nitrifying tandem membrane bioreactor (Bioreactor 1 and Bioreactor 2; van Kessel *et al.*, in preparation), a membrane bioreactor containing an enrichment of “*Ca.* Nitrospira kreftii” (Bioreactor 3; 18), a nitrifying continuous bioreactor (Bioreactor 4; unpublished), an oxygen-limited sequencing batch reactor receiving ammonia and nitrate (Bioreactor 5; unpublished), as well as from the anaerobic compartment of the biofilter of a recirculating aquaculture system [15]. Biomass from soil was harvested by collecting 20 g of soil from an agricultural field (N 51°50'39.4″, E 5°53″8.9″) and either directly processed after resuspension in phosphate-buffered saline (PBS; pH 7.4) or pre-incubated by flooding for one week with demineralized water, either without or with 3.8 mM urea supplied as this was previously shown to increase activity of nitrifiers [29]. Microorganisms were extracted from the soil by sonication (6 × 30 s, with 1 min pauses, on ice, 30 W) using a Sonifier B-12 Probe Sonicator (Branson Sonic Power, Danbury, USA), followed by Histodenz (80% w/v) density centrifugation [30]. To optimize viability of the biomass after sorting, all steps up to cultivation (biomass inhibition, flock disruption, fluorescent labeling, and cell sorting; see below) were performed on the same day as biomass collection. For soil samples, all steps subsequent to biomass extraction and up to cultivation were performed on the same day.

### Biomass inhibition and floc disruption

The obtained biomass suspensions were centrifuged (10 min, speed was optimized for each biomass sample), washed twice with filter-sterilized PBS (pH 7.4) and resuspended in 1 mL PBS. Subsequently, the biomass was incubated in the presence of 1,7-octadiyne (1,7OD), a known inhibitor of the ammonia monooxygenase (AMO), by adding either 10 μL of 10 mM 1,7OD dissolved in dimethyl sulfoxide (DMSO 100 μM final concentration; Sigma Aldrich, Saint Louis, USA) [28] or 10 μL DMSO alone and incubating for 30 min at room temperature (RT). Alternatively, to test the usability of the copper-free tetrazine/trans-cyclooctene ligation, 10 μL 10 mM methyltetrazine-PEG4-alkyne in DMSO (MTH; 100 μM final concentration; Click Chemistry Tools, Scottsdale, USA) or, to test the ability of alkylated fluorescent dyes to directly inhibit the AMO, 20 μL 5 mM 6FAM-alkyne dissolved in DMF (100 μM final concentration; Click Chemistry Tools) were added and incubated for 60 min at RT. After centrifugation (10 min, 10 000 × *g*), biomass was washed 4 times with PBS with vigorous mixing during each washing step by resuspending and/or vortexing to remove traces of the poorly water-soluble inhibitors.

In order to improve the efficiency of the fluorescent labeling and to optimize for the fraction of planktonic cells, flocs were disrupted by a combination of chemical treatment with 0.1% (v/v) Tween 80 or 0.04% (v/v) Tylaxopol and mechanical treatment: using sonication on ice for 1 s, 2 × 2 s, or 10 × 2 s with a Sonifier B-12 Probe Sonicator (Branson Sonic Power) at 30 W, ten times passing the cell suspension through a 0.2 mm needle, rapid shaking for 1 min at 50 Hz in a TissueLyzer LT (Qiagen, Venlo, the Netherlands), or three times passing through a ball-bearing Cell Homogenizer (Isobiotec, Heidelberg, Germany) using a clearance of 6 or 16.

### Fluorescent labeling

The protocol for the Cu(I)-catalyzed azide-alkyne cycloaddition (CuAAC) reaction was described previously [28]. Briefly, 5 mM sodium ascorbate, 5 mM aminoguanidine, and a pre-mixed solution of 100 μM CuSO_4_, 500 μM THPTA, and 5 μM AZDye 488 Azide (Click Chemistry Tools, Scottsdale, USA) or AZDye 488 Azide Plus (Click Chemistry Tools) was added to the obtained biomass from previous steps. Alternatively, to MTH-treated biomass, 10 μM AZ Dye 488 TCO (Click Chemistry Tools) was added. Biomass was incubated for 1 hour and subsequently washed four times with PBS (10 min, 10 000 × *g*) while mixing during each washing step vigorously by resuspending and/or vortexing. Prior to cell sorting, biomass was optionally counterstained with 12 ng/μL Hoechst 34580 for 20 min and washed once with PBS (10 min, 10 000 × *g*) to remove background fluorescence.

### Cell sorting

Flow cytometry and fluorescence-activated cell sorting (FACS) was performed on a BD FACSMelody (Becton Dickinson, Franklin Lakes, USA), equipped with 405, 488, and 561 nm lasers and 8 detectors in a 2–2–4 configuration. The detector for forward scatter (FSC) was equipped with a 0.1 neutral density filter, the detector for side scatter (SSC) with a 488/15 BP filter, the detector for green fluorescence (FITC) with a 507 LP dichroic mirror coupled with a 507/32 BP filter, and the detector for blue fluorescence (V450) with a 448/45 BP dichroic mirror. The FACS was operated using FACSChorus software (v3; Becton Dickinson) for automated instrument calibration and setup using CS&T beads (Becton Dickinson), and Accudrop beads (Becton Dickinson).

Both flow cytometry recordings and cell sorts were performed at a flow rate of 1 to reduce shear stress on cells. Thresholding was performed based on the FSC signal. After recording 10 000–100 000 events, gates were defined in the SSC-FITC plots of control samples allowing for a false positive population <0.5%. Gated populations were sorted into Tissue Culture Plates (non-treated, 96 wells; VWR, Radnor, USA) by sorting between 1 and 1000 events per well, containing 200 μL mineral autotrophic medium (see below) with an ammonium concentration ranging from 200–1000 μM NH_4_Cl and optionally 20 μM NaNO_2_. The well plates were sealed with parafilm and placed in an air-tight, resealable plastic bag containing moisturized tissue to prevent evaporation from the wells. Flow cytometry plots were prepared using floread.io (Floreada Cytometry).

### Cultivation

Culture media and trace element solutions are summarized in Tables S1 and S2, respectively.

Cultivation was performed in Tissue Culture Plates (96 wells; VWR, Radnor, USA), 5 mL polystyrene round-bottom tubes, or acid-washed Schott bottles (100, 250, or 500 mL). Cultures were maintained at RT, without shaking, and in the dark. Cultures were screened for the production of nitrite and/or nitrate using a Griess diazotization reaction (see below) after 5–7 months. Once activity was observed, cultures were transferred. Active cultures were transferred to bigger volumes (in general a dilution factor of 1:100 was used) and/or refed with 0.2–1 mM NH_4_Cl, and regularly (2–3 weeks interval) screened for ammonium consumption using Nessler's reagent (see below) and nitrite and/or nitrate production using nitrite/nitrate test strips (MQuant Nitrate strips, Merck, Darmstadt, Germany).The activity of the cultures is affected by the experimental procedure, as labeling and subsequent sorting did result in a decrease in activity.

### Colorimetric assays

Ammonia was quantified using a modified *o*-phthalaldehyde assay mixing 150 μL reagent (40 mM *o*-phthalaldehyde in 400 mM K_x_H_x_PO_4_ [pH 7.3], 10% [v/v] ethanol, 0.05% [v/v] β-mercaptoethanol) with 10 μL sample [31]. After incubation (20 min, RT), absorbance was measured at 405 nm using the SpectraMax190 plate reader (Molecular Devices, San Jose, USA). Nitrite was quantified using a modified Griess diazotization reaction; 50 μL reagent A (1% sulfanilic acid [w/v] in 1 M HCl), 50 μL reagent B (0.1% [w/v] N-(1-naphthyl)ethylenediamine [NED]) and 100 μL sample were mixed. After incubation (10 min, RT), absorbance was measured at 540 nm, after which nitrate was quantified within the same sample by reducing nitrate to nitrite by adding 6.9 mM VCl_3_ and incubation (30 min, 60°C). Absorbance was remeasured and nitrate concentration was determined by correcting for the initial nitrite concentration [32]. Semi-quantitative measurements of ammonia were performed with Nessler’s reagents (Merck, Darmstadt, Germany) and of nitrite and nitrate with MQuant Nitrate strips (Merck).

### Deoxyribonucleic acid extraction and polymerase chain reaction

Simultaneous to the processing of biomass for cell sorting, pellets of 50 mL bioreactor or RAS filter samples, or 0.25 g agricultural soil were stored at −20°C for DNA isolation. DNA was extracted with the DNeasy PowerSoil Pro Kit (Qiagen, Hilden, Germany) according to the manufacturer’s protocol except for the bead beating step, which was performed in a TissueLyzer (1 min, 50 Hz; Qiagen). Sorted cultures (25 mL) were centrifuged (20 min, 10 000 × *g*, 4°C) in glass centrifuge tubes. Subsequently, pellets of the cultures were divided for fixation (see below) and DNA extraction using DNeasy Blood & Tissue Kit (Qiagen) performing the protocol for Gram-negative bacteria. Extracted DNA was quantified using the Qubit dsDNA HS Assay (Thermo Fisher Scientific, Waltham, USA).

End-point polymerase chain reactions (PCRs) were performed with 10 ng template DNA, 500 nM forward and reverse primers and 1X PerfeCTa SYBR Green SuperMix (Quantabio, Beverly, USA) on a LabCycler Gradient (SensoQuest, Göttingen, Germany) according to following protocol: initial denaturation at 96°C (5 min), 35 cycles of denaturation (30 s, 96°C), annealing (30 s, 52 or 55°C) and elongation (60 s, 72°C), final elongation (10 min, 72°C), and cooling at 4°C. Primer details and annealing temperatures can be found in Table S3. PCR products were separated by agarose gel electrophoresis (1.5% agarose in TBE, 80 V, 50 min). PCR products were isolated with the GeneJET PCR Purification kit (Thermo Fisher Scientific) and quantified using the Qubit dsDNA HS Assay.

### Sanger sequencing and phylogeny

Purified PCR products were directly sent for Sanger sequencing (Baseclear, Leiden, the Netherlands). The obtained 16S ribosomal RNA (rRNA) gene sequence lengths were 1391 basepairs (bp) for *Nitrosomonas* and between 1047 and 1049 bp for *Nitrospira*. The obtained *amoA* sequences ranged from 182–756 bp. Sequences have been deposited in the European Nucleotide Archive (ENA; project PRJEB81149). Reference nucleotide sequences for the 16S rRNA gene and amino acid sequences for AmoA were obtained from NCBI [33]. The reference sequences were processed along with the sequences obtained in this study through the default phylogeny.fr pipeline [34]; MUSCLE (v 3.8.31) was used for sequence alignment [35], alignments were filtered with GBlocks (v 0.91b, [36], and maximum likelihood was applied to reconstruct the phylogeny using PhyML (v 3.1/3.0 aLRT) [37]. Phylogenetic trees were visualized and annotated with iTOL (v5) [38].

### Fluorescence *in situ* hybridization and microscopy

Samples for fluorescence microscopy were first fixed with 3% (v/v) formaldehyde (60–90 min, 4°C), washed twice with PBS and stored in a 1:1 PBS:ethanol mixture at −20°C. Fixed cells were either mounted directly with Vectashield Antifade containing DAPI (Vector Laboratories, Newark, USA) or after performing fluorescence *in situ* hybridization (FISH). Hybridizations were performed at a formamide concentration of 35% using reaction conditions described elsewhere [39]. Probes used in this study are listed in Table S4. Microscopic images were acquired on a confocal laser scanning microscope (Sp8x, Leica Microsystems, Mannheim, Germany), equipped with a 405 nm diode and a pulsed white-light laser (470–670 nm). Images were acquired with 100× oil objective at 8-bit depth, and processed with FIJI [40].

## Results

### Protocol optimization and description

We aimed to establish a targeted and high-throughput cultivation approach for (complete) ammonia-oxidizing bacteria by means of combining specific CuMMO fluorescent labeling with cell sorting. To identify and alleviate potential bottlenecks hampering an effective cultivation approach, we performed preliminary tests on biomass from a tandem membrane bioreactor. First, nitrifying bacteria often grow in flocs or biofilm, resulting in co-sorting of different nitrifiers and attached heterotrophs if no floc disruption methods are applied upfront. Second, the addition of copper, especially the Cu(I) required as essential catalyst for the CuAAC reaction, necessitates minimizing its toxicity effects on bacteria [41]. Third, the sorting process needs to be selective to yield target cells only and needs to be gentle to maintain viability. Finally, a suitable growth medium needs to be selected that maximizes the retrieval rate of nitrifying cultures from the respective sample.

Physical floc disruption methods resulted in a larger fraction of planktonic cells than chemical disruption, but combining both methods may have a synergistic effect (Fig. S1). Sonication and ball-bearing homogenization resulted in the highest recovery rate of planktonic cells. Interestingly, the number of sonication pulses only marginally improved the planktonic fraction, whereas a smaller clearance during homogenization increased the number of planktonic cells and decreased average floc size. In contrast, passing the biomass through a needle or disruption using the TissueLyzer resulted in negligibly more planktonic cells compared to the non-treated control sample. Chemical detergents like Tween-80 and Tylaxopol, on the other hand, had no effect on their own, but slightly improved the fraction of planktonic cells after sonication with multiple pulses and after needle passage. Most disruption methods had a minimal influence on the ammonia-oxidizing activity of the biomass (Fig. S2). Only ball-bearing homogenization substantially decreased the activity indicating too harsh disruption of the biomass leading to cell lysis.

A larger decrease in ammonia-oxidizing activity was, however, observed during prolonged exposure to the tested inhibitors and auxiliary CuAAC chemicals (Fig. S3). Notably, ascorbate drastically decreased activity, especially in combination with CuSO_4_, which results in the formation of the Cu(I) cations required as reaction catalyst. Notably, while the addition of aminoguanidine can be omitted from the reaction without decreasing signal intensity, it partially rescued this loss of activity (Fig. S4). Interestingly, (potential) CuMMO inhibitors like 1,7-OD and other *n*-terminal alkynes showed an initial decrease in activity that, however, quickly recovered. It should be noted that large biological differences were observed, which may be explained by the heterogeneity of the biomass.

The observed partial reduction in activity upon treatment with CuAAC chemicals prompted us to investigate other Click labeling strategies. The CuAAC reaction was, however, found to be the only available Click chemistry compatible with the CuMMO enzyme family, but its efficiency could be improved by substituting the standard azide dyes with copper-chelating azides (Fig. S5). Such dyes with a copper-chelating motive like Azide 488 Plus have faster second-order reaction kinetics and resulted in greatly improved fluorescence signals, resulting in a better separation of positive and negative populations during FACS. An alternative strategy involving initial inhibition with methyltetrazine-alkyne followed by a tetrazine-TCO ligation with a 488-TCO fluorophore yielded high background signals in the negative control. We also tested direct labeling of the CuMMO with an alkyne-containing fluorophore (6FAM-alkyne), but no fluorescent signal was obtained using this strategy, presumably due to steric hindrance in the active site.

In the end, we wanted to confirm whether the entire protocol, from floc disruption with tylaxopol and sonication, to labeling with CuAAC and cell sorting, allowed the retrieval of viable cultures. To assess this, a bulk sort containing mostly fluorescently labeled single cells and small cell clusters was incubated with 1 mM ammonia. Samples that underwent the entire protocol but were not sorted fully oxidized 1 mM ammonia to nitrate within 13 days, and within 2–3 months for the bulk-sorted fractions. Thus, the labeling protocol resulted in a viable population of ammonia-oxidizers.

### Protocol performance in complex communities

The optimized protocol was applied to samples from five bioreactors, one natural habitat (agricultural soil), and one engineered system (RAS biofilter). These samples contained different combinations of AOB, AOA, and clade A and B comammox *Nitrospira* (Fig. S6). Two different cultivation media were selected for most habitats to assess the effect of culture medium on the number and types of nitrifiers obtained.

**Figure 2:**
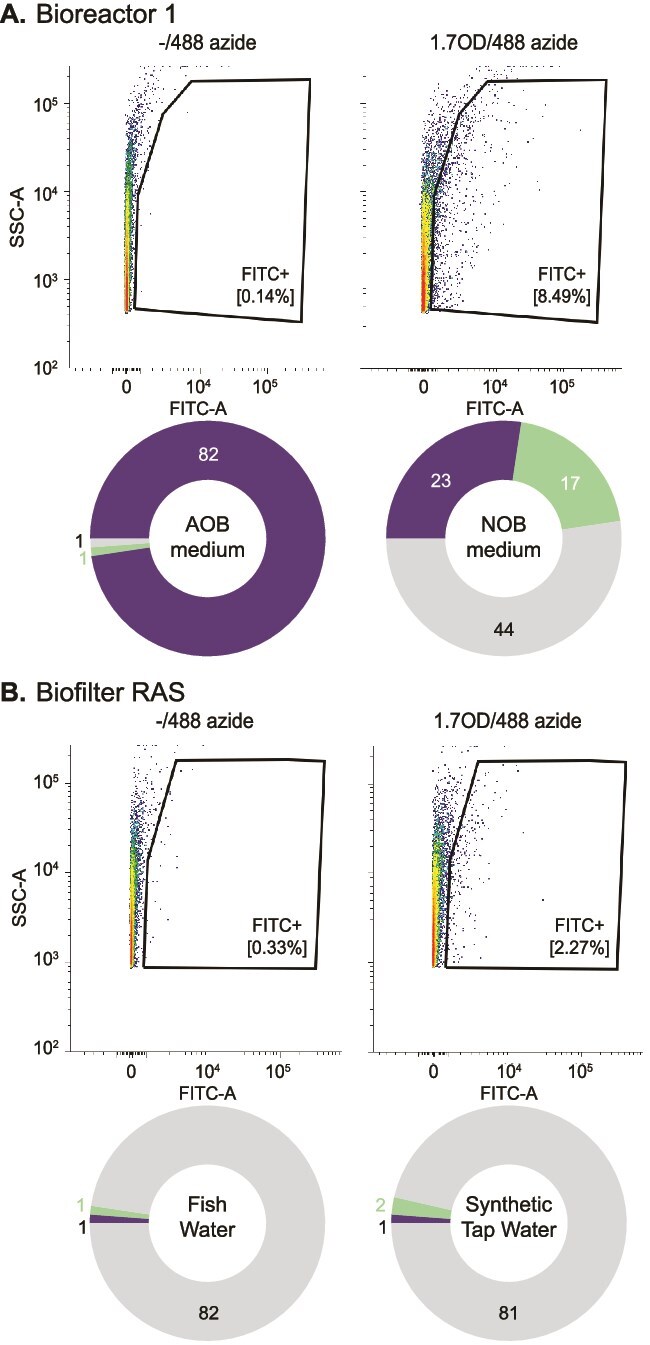
Fluorescence-activated cell sorting of labeled ammonia-oxidizing bacteria. Biomass samples from bioreactor 1 (A) and the RAS biofilter (B) were incubated without 1,7-octadiyne (1,7OD) but subjected to the CuAAC reaction with AZDye488 to set the gates, and with 1,7OD followed by the same CuAAC reaction for sorting positive events. The doughnut charts indicate the number of inactive (gray) or active wells producing nitrite (blue) or nitrate (green) after sorting into 96-well plates containing the indicated types of mineral medium.

All samples showed fluorescent signals after labeling during flow cytometry (Fig. 2, Fig. S7, Table S5). Only for the soil sample, a preincubation step with urea was required to see labeled cells, presumably due to a low abundance of AMO enzymes in this ammonium-limited habitat (Fig. S8). Using the control samples of the Click procedure, sorting gates were carefully constructed that minimized the number of false-positive events sorted (Fig. 2, Fig. S7, Table S5). In each row in the receiving 96-well cultivation plates, wells were inoculated with an increasing number of events (1, 3, 10, 30, 100, 300, 1000) to account for differences in cellular viability after sorting. Subsequently, the 96-well plates were incubated at RT 5–7 months before testing for nitrite or nitrate formation.

The number of retrieved active wells varied greatly across samples, but also within samples between the different cultivation media (Fig. 2, Fig. S7, Table S5). The largest number of active wells (*n* = 83) was obtained from Bioreactor 1 of the tandem membrane bioreactor and almost exclusively nitrite-producing wells were observed in AOB medium (*n* = 82), whereas incubations in NOB medium yielded nitrite (*n* = 23) as well as nitrate-producing wells (*n* = 17). Bioreactor 2 showed fewer active wells in comparison, comprising only nitrate-producing wells (*n* = 19) in NOB medium and one single nitrite-producing well in AOB medium. Bioreactor 3 only yielded active, nitrate-producing wells (*n* = 18), all in NOB medium. Bioreactor 4 showed various nitrate-producing wells (*n* = 10) in groundwater medium as the only medium tested. Using biomass from Bioreactor 5, only one single active well was observed in synthetic tap water medium. The RAS biofilm and biomass extracted from soil only showed few nitrite and nitrate-producing wells in the tested media (Fig. S7).

### Identification of nitrifying cultures

Active wells were upscaled to larger volumes; in general, active cultures were diluted 100 times with fresh medium. Identity screening was performed through *amoA* PCR and Sanger sequencing. For all tested cultures, either clade A *amoA* or AOB *amoA* PCR products were obtained. The absence of AOA *amoA* in the active wells was expected since 1,7-octadiyne does not label AOA [28]. The absence of clade B *amoA* PCR products may be attributed to inapt culturing conditions, as so far, no enrichments of clade B comammox have been described. Furthermore, only the RAS biofilm and soil sample used here contained clade B comammox. Based on the sequencing results, duplicate cultures were removed from the culture collection, prioritizing cultures obtained from wells that received the lowest number of events to ensure minimal culture complexity.

**Figure 3:**
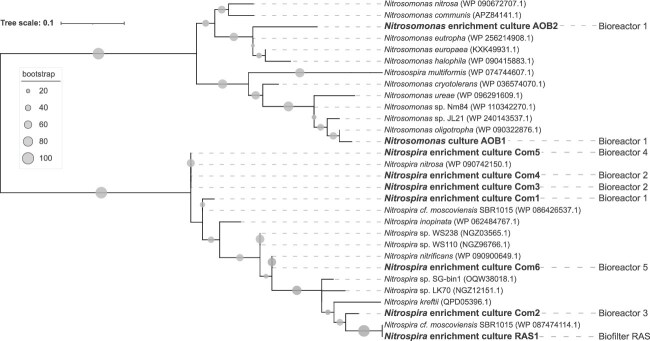
Maximum likelihood AmoA protein-based phylogenetic tree of selected representatives of ammonia-oxidizing *Nitrosomonadaceae* and clade a comammox *Nitrospira*. Sequences of the (enrichment) cultures obtained in this study are indicated in bold, biomass source is indicated to the right. Bootstrap support values of 100 replicates are represented by the size of the circles.

In the end, few habitats yielded multiple but most only a single nitrifying culture (Fig. 3). From Bioreactor 1, two AOB cultures and one comammox strain, and from Bioreactor 2, two comammox strains were obtained. Bioreactor 3, 4, and 5 and the RAS biofilter yielded one comammox strain each. For agricultural soil, we did not manage to successfully upscale any culture.

Phylogenetic analysis of the obtained *amoA* sequences revealed that the obtained cultures belong to *Nitrosomonas* cluster 6a or 7, or to comammox *Nitrospira* clade A. Three of the seven obtained comammox strains clustered with *Nitrospira nitrosa*, one with *Nitrospira nitrificans*, two more distantly with *Candidatus* Nitrospira kreftii, and one with no cultured representative. These findings were corroborated by 16S rRNA data with all *Nitrospira* 16S rRNA gene sequences clustering within lineage II *Nitrospira* and closely together with known comammox species (Fig. S9). The two AOB *amoA* sequences affiliated with *Nitrosomonas* clusters 6a and 7 were most similar to *Nitrosomonas oligotropha* and *Nitrosomonas eutropha*, respectively. Likewise, the 16S rRNA gene sequence obtained using general 16S rRNA primers from one of these cultures (AOB1) clustered with *N. oligotropha* (Fig. S10)*,* whereas for the other AOB culture (AOB2) a mixed 16S rRNA PCR product was obtained.

**Figure 4:**
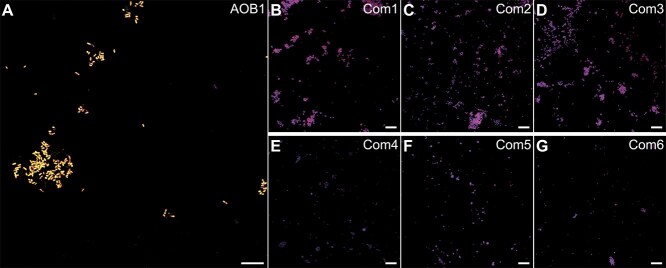
FISH of the active cultures obtained after targeted cell sorting. (A) Culture *Nitrosomonas* AOB 1 hybridized with nm-OL-703 (red) and Eub388mix (green); (B–G) enrichment cultures of (B) *Nitrospira* Com1, (C) *Nitrospira* Com2, (D) *Nitrospira* Com3, (E) *Nitrospira* Com4, (F) *Nitrospira* Com5, (G) *Nitrospira* Com6, hybridized with Ntsp0662 and Ntsp0712 (red).

FISH analyses of the active cultures indicated a varying degree of enrichment (Fig. 4). One AOB culture from Bioreactor 1 and one comammox culture from the RAS biofilter lost activity during upscaling, and sufficient biomass for FISH could not be obtained. While five of the six remaining comammox *Nitrospira* cultures were highly enriched, enrichment culture Com4 was found to contain only a small fraction of *Nitrospira* cells. Notably, for the AOB1 culture, the *Nitrosomonas* cluster 6a + b-specific probe Nm-OL-703 hybridized to all cells counterstained by the general bacterial EUB338 probe mix (Supplementary Table S4). Additionally, the absence of heterotrophic growth in both LB-Miller and 10-fold diluted LB-Miller mineral medium, the single 16S rRNA sequence obtained with direct Sanger sequencing after a PCR employing general bacterial primers, and the absence of PCR products when using archaeal 16S rRNA gene primers indicate an axenic culture of a novel AOB species.

## Discussion

Nitrifiers are known for their recalcitrance to cultivation and many ammonia oxidizers are only available as enrichment cultures. The isolation of many of these cultures we have available today, took years of cultivation efforts (see, for instance, 17,18,19,20) and often required the combination of conventional cultivation techniques with single-cell isolation methods (e.g. 2,24,25). Here, we developed and optimized a cultivation approach targeting ammonia-oxidizing microorganisms that combines Cu-MMO labeling using Click chemistry with fluorescence-activated cell sorting to selectively isolate nitrifiers and thereby overcome the long cultivation times. The protocol was established by optimization of all individual steps of the workflow toward selectivity and viability. Disruption of flocculant biomass performed best with mild sonication in combination with chemical detergents (Fig. S1). Obtaining fluorescently labeled cells from soil was found to require a pre-incubation step with urea, as applying the protocol to the native soil yielded insufficient signal (Fig. S8). Despite the negative effects of copper toxicity on the nitrifying activity of the biomass, the addition of copper remains essential for CuAAC, and the tested copper-free alternative labeling approaches did not yield labeled cells (Fig. S5). Nevertheless, viable cultures could be obtained from biomass that was subjected to the CuAAC reaction. However, large differences in culture retrieval rates were found between samples and different cultivation media. Multiple nitrifiers were retrieved from some lab-scale bioreactors, whereas natural biomass sources only yielded a single nitrifying culture (Figs. 2 and S7). In the end, we managed to obtain one axenic and one enriched AOB culture and seven comammox *Nitrospira* enrichment cultures (Figs. 3, S9, and S10).

Our targeted cultivation approach offers the advantage of relatively high throughput. By selecting for single cells, it allows obtaining multiple nitrifiers from the same habitat in parallel. Compared to more traditional batch or continuous cultivation approaches that focus on one single habitat [42] and oftentimes retrieve one single isolate or enrichment culture [43], we managed to obtain seven comammox *Nitrospira* enrichment cultures in this study, which represents the largest increase in comammox cultures reported so far. Furthermore, the recovery of multiple phylogenetically diverse ammonia-oxidizing cultures from the same habitat gives the unique opportunity to study the niche differentiation of different guilds and their interactions, as has been done previously for NOB isolated from the same WWTP [44]. The niche differentiation and coexistence of different nitrifying guilds remains elusive and more studies with synthetic co-cultures [45] using nitrifiers from the same habitat could greatly enhance our knowledge.

Notwithstanding, several challenges still need to be overcome, for instance, the toxicity of the CuAAC reaction, the poor cultivability and retrieval rates from natural environments such as soil, and the suboptimal culture retrieval rates in general. The usage of Cu(I) is virtually impossible to circumvent since only *n*,*n*-terminal alkadiynes appear to be able to enter the CuMMO active site, whereas larger molecules are likely sterically hindered. Besides CuAAC, alkadiynes offer the possibility to use Palladium-mediated cross-coupling [46] or Ruthenium-Catalyzed Azide Alkyne Cycloaddition [47] to partially mitigate copper toxicity. If able to react with the CuMMO active site, other inhibitors than alkadiynes would allow a larger spectrum of potential Click reactions. For instance, small bifunctional hydrocarbons containing one alkyne moiety in combination with either an alkene or azide functional group could provide new possibilities such as Light-induced Click reactions [48] or Strain Promoted Azide Alkyne Cycloadditions [49], respectively.

The poor retrieval rates of nitrifying cultures from certain environments may be explained by applying different growth media or cultivation conditions (e.g. aerobic *vs*. hypoxic) after sorting compared to the original sample. Ideally, the cultivation medium should mimic the conditions of the natural environment as closely as possible [50]. However, for the RAS biofilter, using filtered RAS water instead of artificial mineral medium did not increase culture retrieval. For soil, finding a suitable growth medium may even be more complex, as bacteria are transferred from a solid matrix to liquid medium [51]. Preculturing at similar conditions as applied after sorting might yield more active cultures but will likely bias the culture composition depending on the applied conditions. Lastly, culture heterogeneity may result in only few cells within a population being culturable [52]. Therefore, an additional marker that senses cellular activity such as BONCAT [27], THRONCAT [53], or redox dyes such as Redox Sensor Green [54] could potentially further enhance retrieval rates.

Beyond the nitrifiers obtained in this study, we note that this cultivation approach could be applied to more environments and bacteria carrying other members of the CuMMO family. Foremost, habitats dominated by clade B comammox bacteria such as some DWTP filters [55, 56] and certain black and forest soils [57, 58] would be of high interest, as to date no clade B enrichment culture is available. Beyond nitrifiers, this method is also applicable to methanotrophs and other short-chain alkane oxidizers and could, for instance, be used as a tool to isolate yet elusive anaerobic methanotrophs like the *Methylomirabiliceae* [59]. Several clades within the CuMMO family still lack cultured representatives and experimental evidence for substrate specificity, such as certain betaproteobacteria carrying uncharacterized *xmoA* sequences [60] and a new cluster of archaeal Cu-*mmoA* sequences [61]. Finally, the potential of combining ABPP with cell sorting to obtain novel isolates extends beyond the CuMMO family, since similar strategies have been conducted to obtain functionally active subpopulations of the gut microbiota [62]. Advances in the development of ABPP probes for other redox-active enzymes could eventually lead to labeling strategies specific to other microbial guilds within the biogeochemical element cycles [63].

In summary, our workflow allows the rapid targeted retrieval of nitrifying cultures and, through parallelization, to obtain distinct ammonia-oxidizers from the same environment for niche differentiation studies. Further optimization of pre-incubation conditions, Click chemistry, medium selection, and viability assessment could still further improve the number of distinct cultures retrieved from a single biomass source. Furthermore, by employing different alkadiynes, the cultivation workflow has the potential to be more broadly applied to comammox *Nitrospira* and AOA, but also to bacteria encoding other family members of the CuMMO protein family. We believe that with further advances in automatization of upstream cultivation plate preparation and downstream activity screening and identification, high-throughput approaches become increasingly valuable in broadening our ever-expanding culture collection.

## Supplementary Material

Blom_et_al_supplementary_information_revised_all_ycae145

## Data Availability

All data generated and analyzed during this study are included in this publication and its supplementary material. Sequences have been deposited in the European Nucleotide Archive under project number PRJEB81149.
