# Supplementary material for: Obtaining complete and canonical ammonia-oxidizing bacteria through specific labeling and cell sorting
Source: ISME Commun. 2025 Feb 8;5(1):ycae145. doi: 10.1093/ismeco/ycae145 (PMC11964087; doi:10.1093/ismeco/ycae145)
Supplement: Blom_et_al_supplementary_information_revised_all_ycae145 [file blom_et_al_supplementary_information_revised_all_ycae145.pdf]

Obtaining complete and canonical ammonia-oxidizing bacteria through specific labelling and cell sorting

Pieter Blom[1], Pascal C. Huizing[1], João P.R.C. de Monlevad [1], Maartje A.H.J. van Kessel [1*], Sebastian Lücker [1*]

Supplementary Material

This file contains:

- Supplemental Figures S1 – S10
- Supplemental Tables S1 – S4

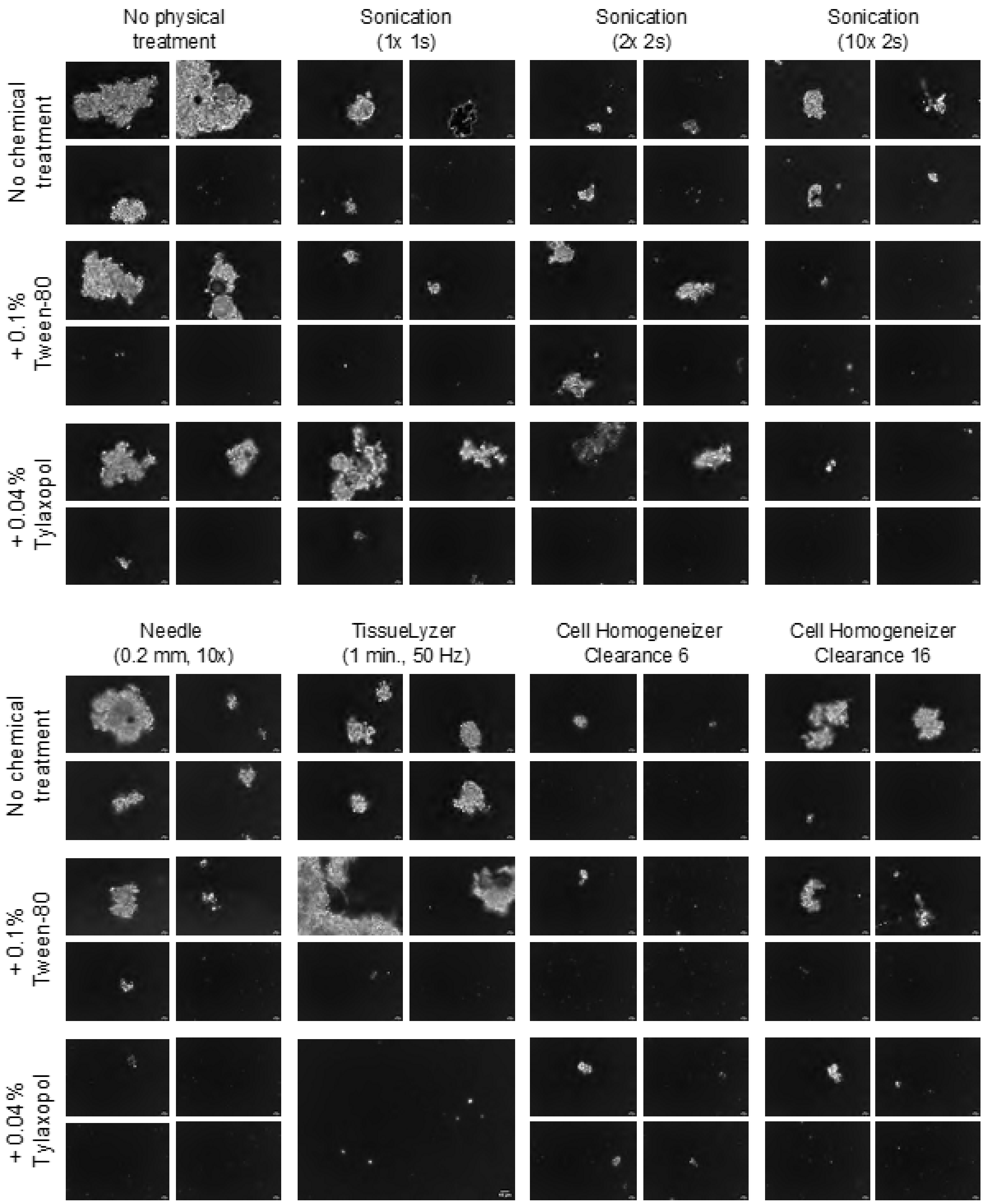

Figure S1: Effects of various disruption treatments on biomass integrity from bioreactor 1. Shown are four representative brightfield micrographs for each combination of physical (untreated control biomass, sonication with different numbers of repetitions, passage through a needle, TissueLyzer, Cell Homogeneizer with different clearances) and chemical treatments (untreated control biomass, Tween-80, Tylaxopol). Note that for the TissueLyzer + Tylaxopol treatment, hardly any biomass was visible and only one micrograph is included.

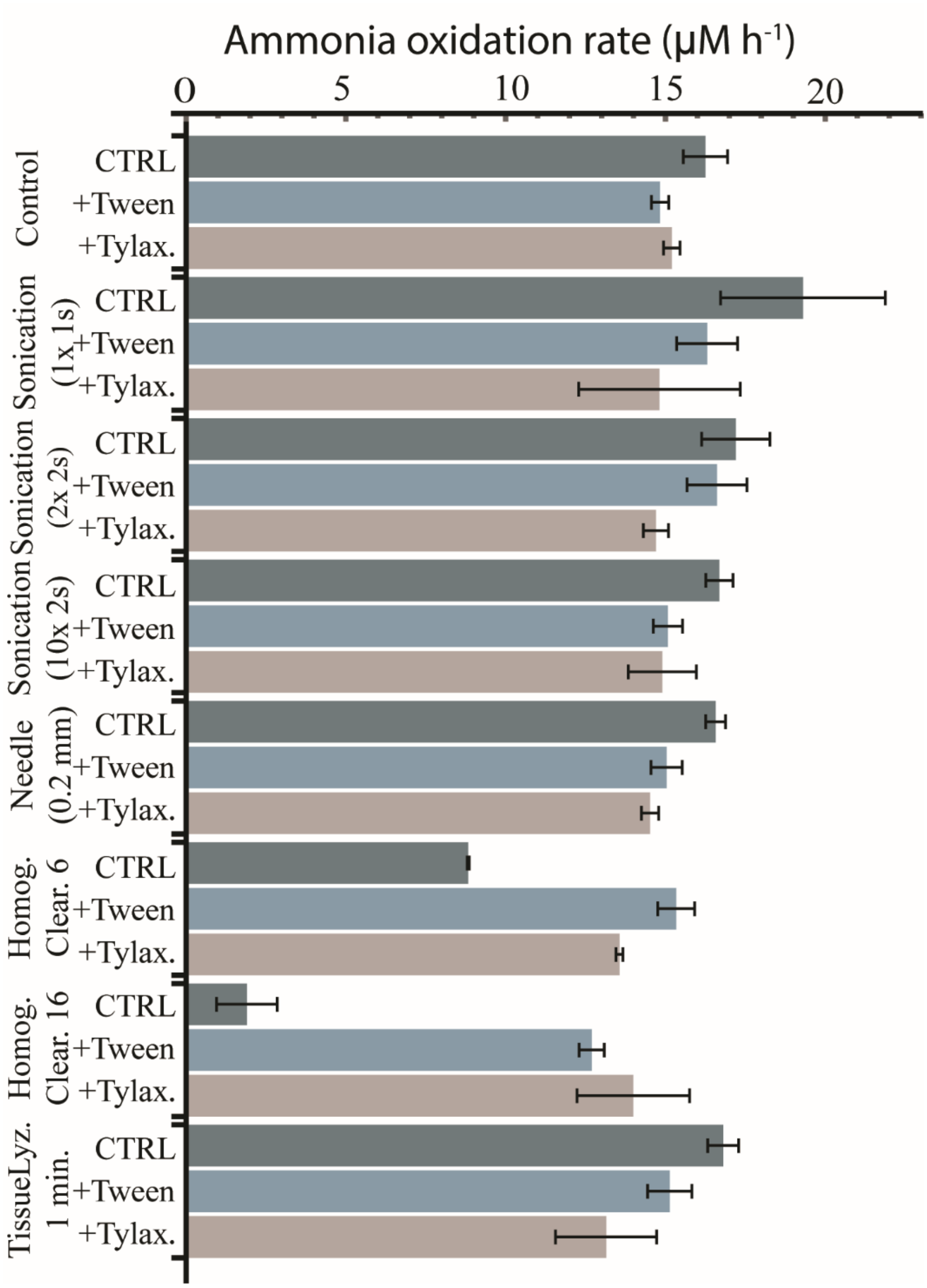

Figure S2: Effect of the tested disruption treatments on ammonia oxidation rates of biomass from bioreactor 1. Bars represent the average rate of three biological replicates for each combination of chemical (untreated control biomass, Tween-80, Tylaxopol) and physical treatments (untreated control biomass, sonication with different numbers of repetitions, passage through a needle, Cell Homogenizer with different clearances, TissueLyzer); error bars represent the standard deviation (n=3).

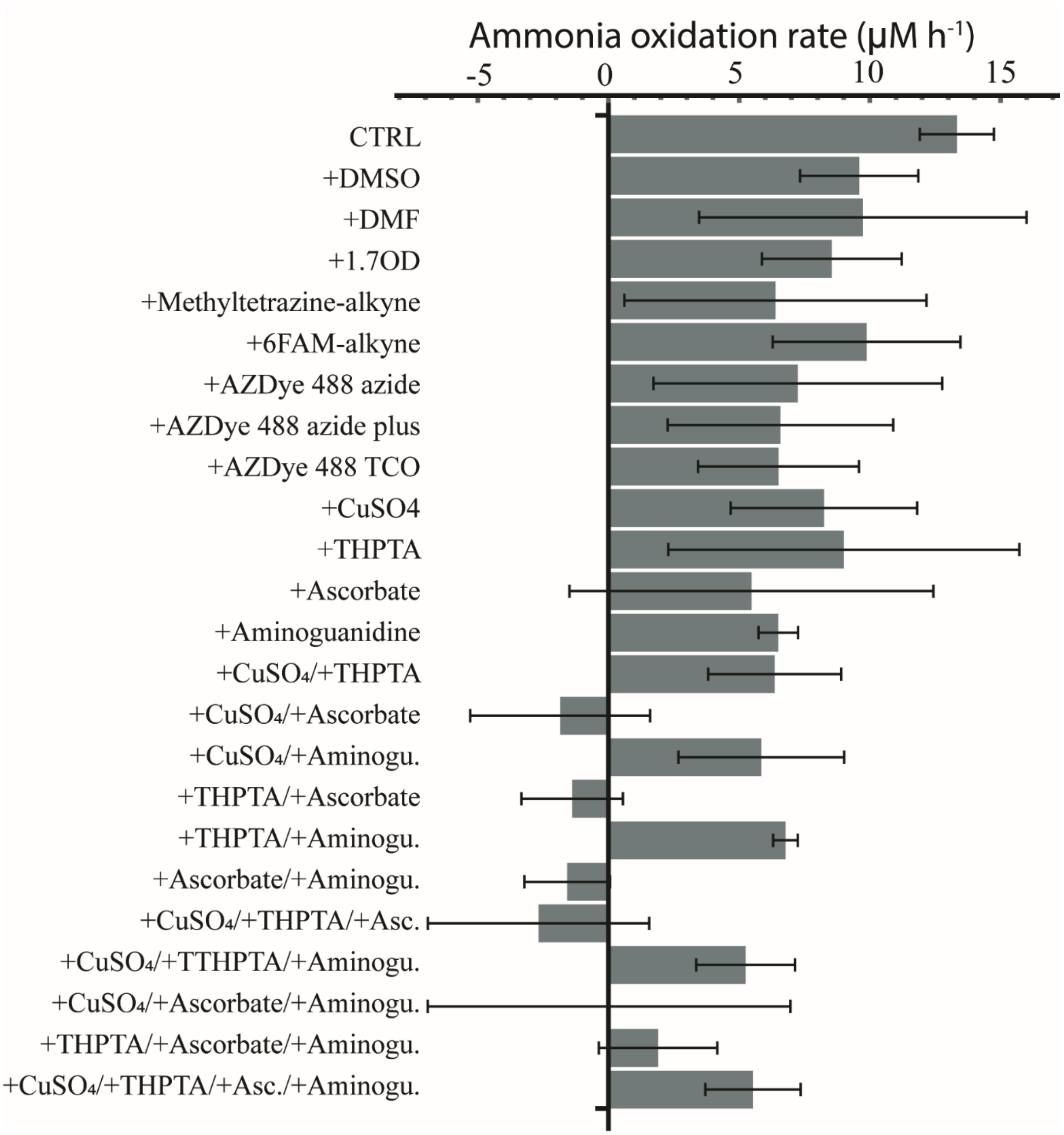

Figure S3: Influence of chemicals used during the CuAAC reaction on ammonia oxidation rates of biomass from bioreactor 1. Bars represent the average of three biological replicates, error bars the standard deviation (n=3). Negative rates indicate the net formation of ammonia.

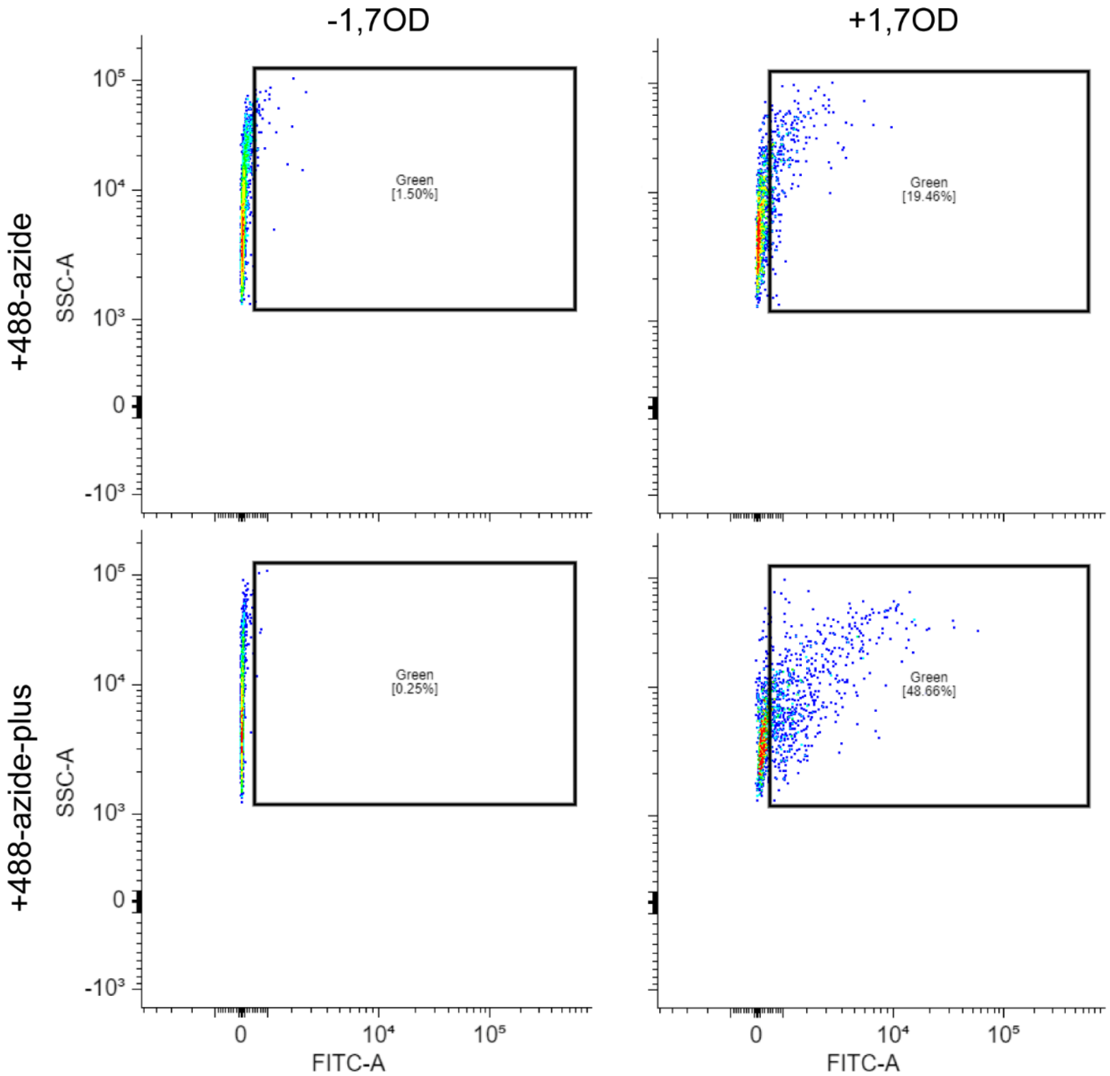

Figure S4: CuAAC reaction efficiency without aminoguanidine addition tested on biomass from reactor 1. Shown are scatter density plots of side scatter (SSC) and green fluorescence (FITC) and set gates, using (top) AZDye 488 Azide and (bottom) AZDye 488 Azide Plus in the CuAAC reaction; incubations were performed without 1,7OD (left) and with 1,7OD (right).

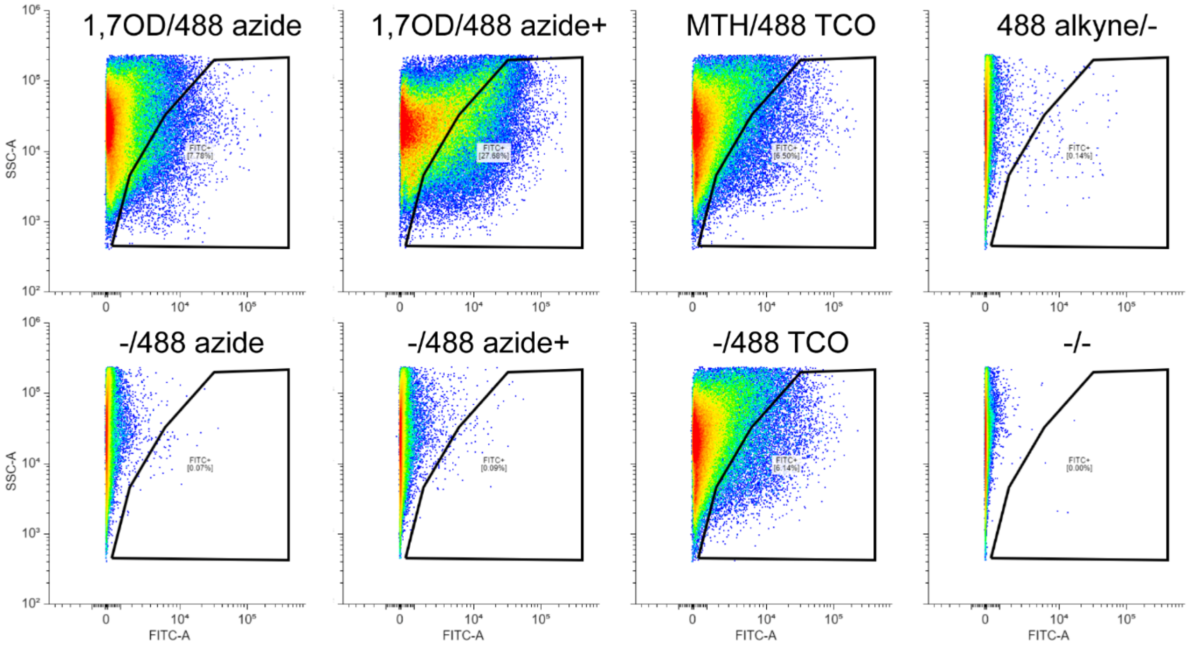

Figure S5: Efficiency and specificity of different AMO-labeling strategies tested on Bioreactor 1 biomass. Shown are scatter density plots of side scatter (SSC) and green fluorescence (FITC); the final gate constructed based on the controls is included in all plots. Biomass was incubated (first column) with (top) and without (bottom) 1,7OD followed by the CuAAC reaction with AZDye 488 Azide; (second column) with (top) and without (bottom) 1,7OD followed by the CuAAC reaction with AZDye 488 Azide Plus; (third column) with (top) and without (bottom) MTH followed by the CuAAC reaction with AZDye 488 TCO; (fourth column) with (top) and without (bottom) AZDye 488 Alkyne instead of 1,7OD and without subsequent CuAAC reaction.

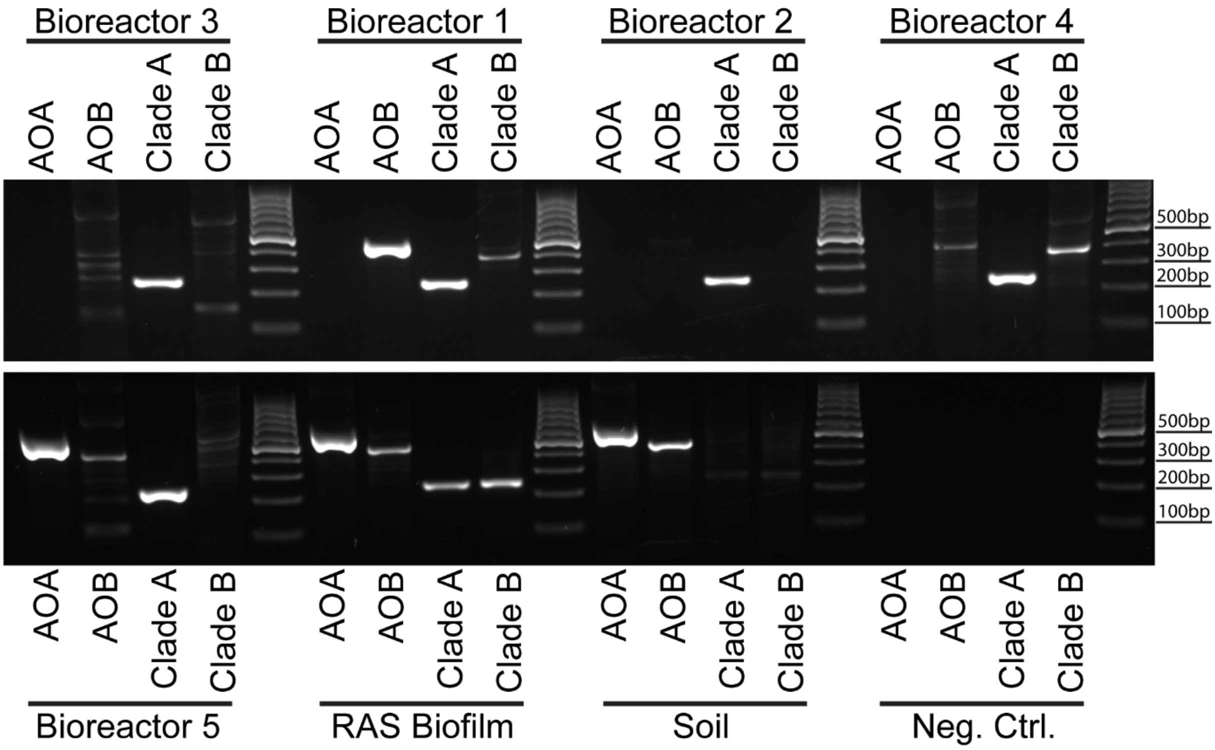

Figure S6: *AmoA* gene-targeted end-point PCR testing for the presence of AOA, AOB, and clade A and clade B comammox *Nitrospira* in all biomass samples. PCR products are expected at 635 bp for AOA, 491 bp for AOB, and 235 bp for both clade A and clade B comammox *Nitrospira*.

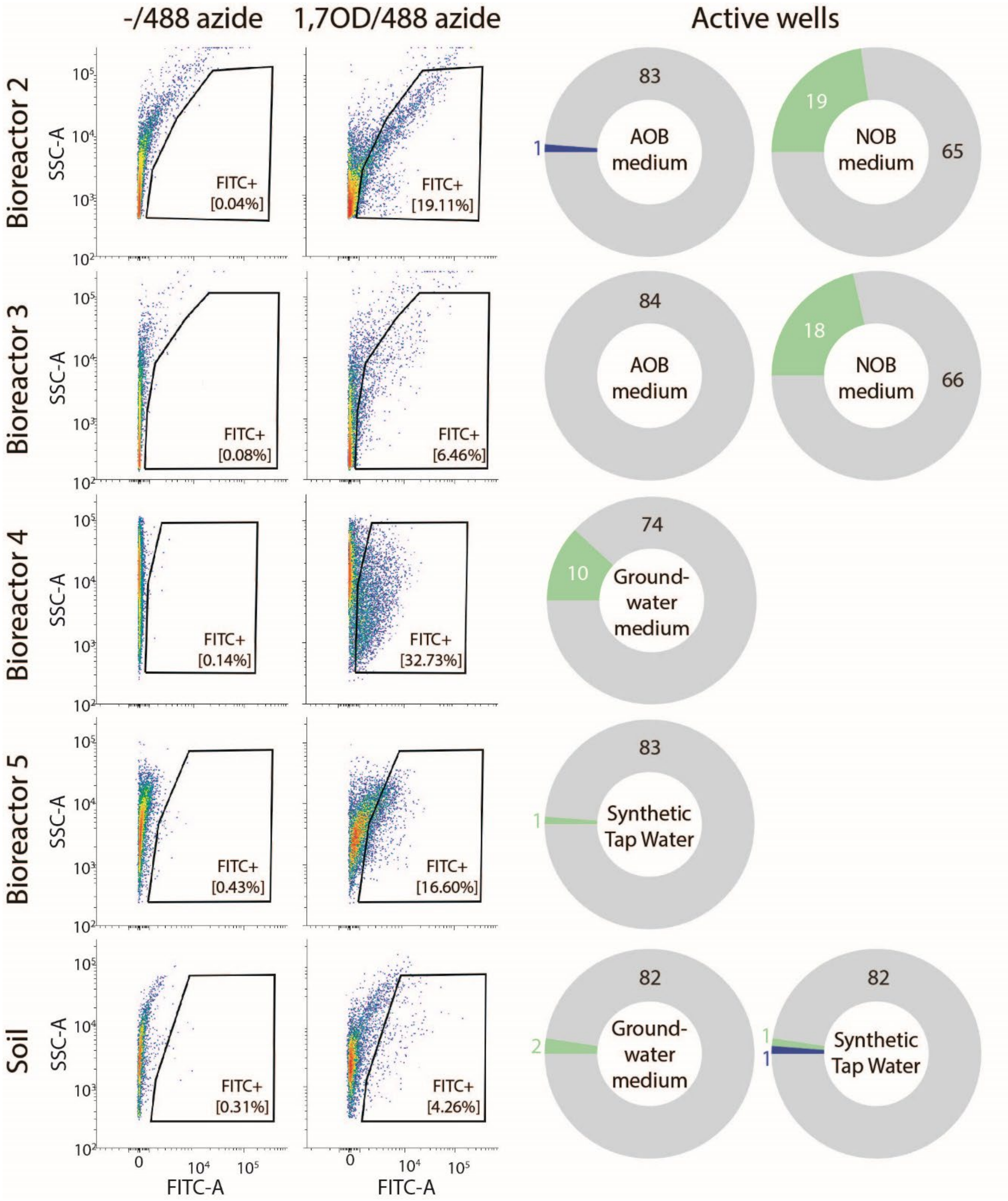

Figure S7: Fluorescence-activated cell sorting of labeled ammonia-oxidizing bacteria. Biomass samples from (rows; from top to bottom) Bioreactor 2, Bioreactor 3, Bioreactor 4, Bioreactor 5, and soil after urea addition. Samples were incubated without 1,7-octadiyne (1,7OD) but subjected to the CuAAC reaction with AZDye488 to construct the gates (first column), and with 1,7OD followed by the same CuAAC reaction for sorting positive events (second column). The doughnut charts (rightmost columns) indicate the number of inactive (gray) or active wells producing nitrite (blue) or nitrate (green) after sorting into 96-well plates containing the indicated types of mineral medium.

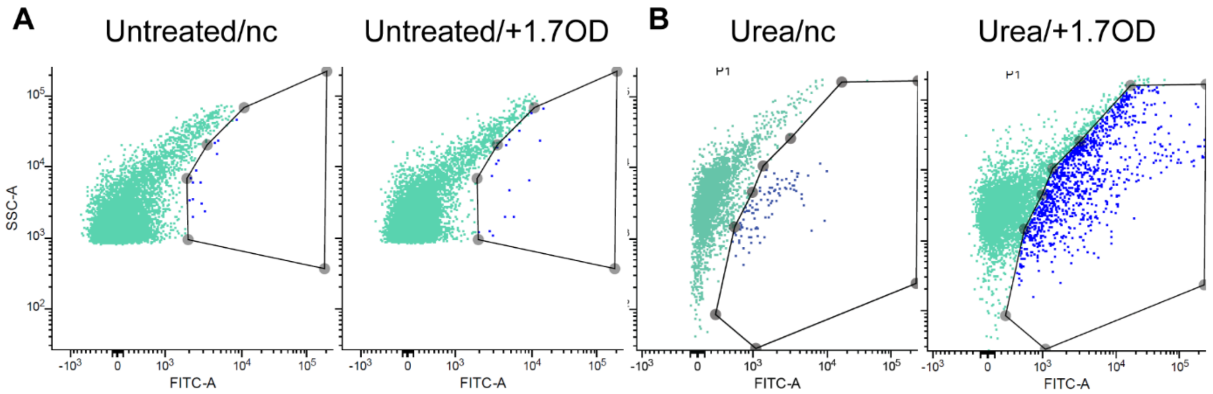

Figure S8: Labeling efficiency of soil biomass with and without urea preincubation. Shown are scatter density plots of side scatter (SSC) and green fluorescence (FITC), and the constructed gates (A) without pretreatment and (B) following urea pretreatment; incubations were performed (left) without and (right) with 1,7OD.

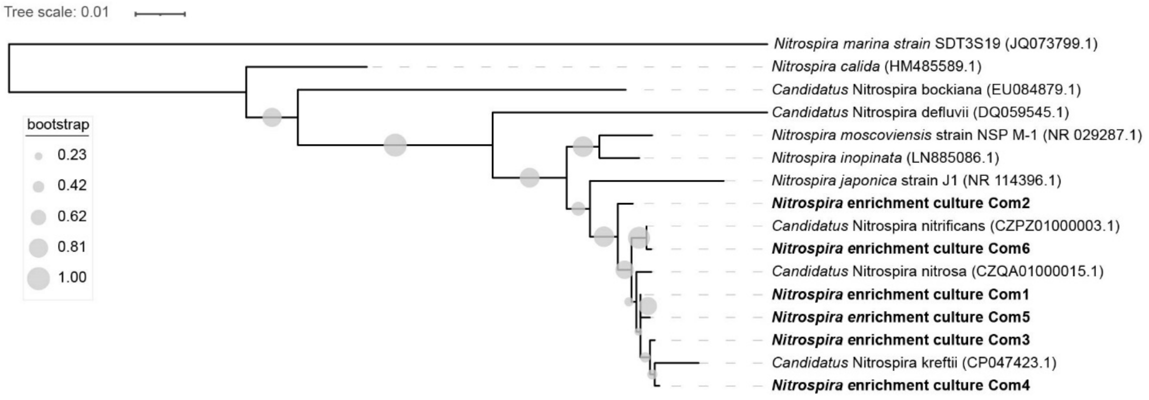

Figure S9: 16S rRNA gene-based maximum likelihood phylogenetic tree showing the affiliation of the obtained *Nitrospira* cultures. Included are sequences of selected genus *Nitrospira* representatives and the enrichment cultures gained in this study (in bold, 1048 base pairs). Bootstrap support values of 100 replicates are represented by the size of the colored circles.

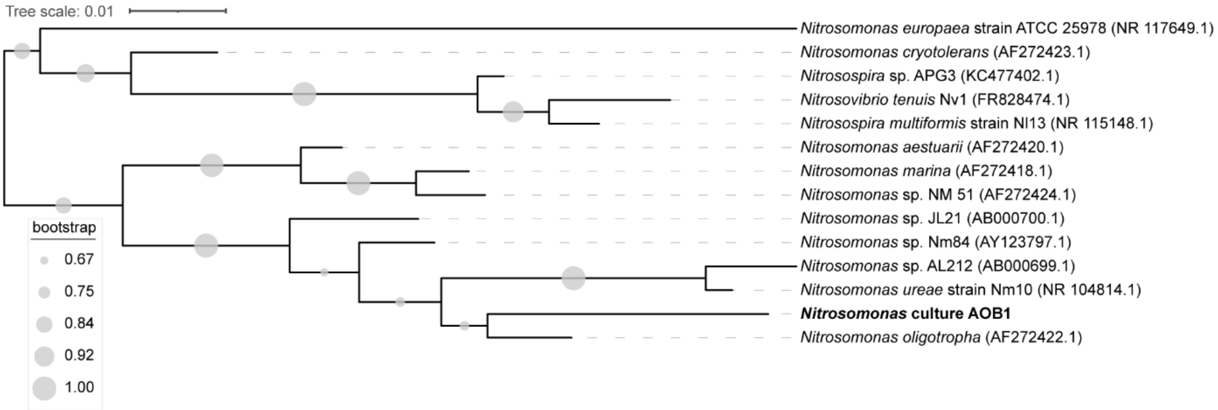

Figure S10: 16S rRNA gene-based maximum likelihood phylogenetic tree showing the affiliation of the obtained *Nitrosomonas*. Included are sequences of selected family *Nitrosomonadaceae* representatives and the culture obtained in this study (in bold, 1391 base pairs). Bootstrap support values of 100 replicates are represented by the size of the colored circles.

Table S1: Composition of culture media used after sorting.

Medium	NaCl (mM)	KCl (mM)	$CaCl_2$ (mM)	$MgSO_4$ (mM)	KH_2PO_4 (mM)	$KHCO_3$ (mM)	HEPES (mM)	TE1# (%)	TE2# (%)	TE3# (%)	Reference
AOB	10	1	1	0.4	0.4	5	-	0.1	0.1	-	1
NOB	8.6	-	0.17	0.2	1.1	1	10	0.1	0.1	-	2
Synthetic Tap Water	1	-	2	0.81	0.21	1	10	0.1	0.1	-	3
Groundwater	0.63	-	0.68	0.41	0.021	1	10	0.1	-	0.1	4
Fish water*	-	-	-	-	-	-	10	-	-	-	5

* Fish water was directly collected from the anaerobic compartment of the RAS biofilter and filter-sterilized through a 0.2 µm filter.

# The composition of trace elements solutions TE1, TE2, and TE3 is given in **Table S2**.

Table S2: Composition of trace elements solutions.

Solution	$FeSO_4$ (mM)	$ZnSO_4$ (mM)	$CoCl_2$ (mM)	$MnCl_2$ (mM)	$CuSO_4$ (mM)	Na_2MoO_4 (mM)	$NiCl_2$ (mM)	Na_2SeO_4 (mM)	H_3BO_3 (mM)	$CeCl_3$ (mM)	NTA (g/L)	Reference
TE1	18	-	-	-	-	-	-	-	-	-	10	-
TE2	-	1.5	1	5	1	0.91	1.5	0.58	2.3	0.64	5.5	6
TE3	-	0.77	0.004	8.1	0.32	0.083	0.39	0.53	8.1	0.67	15	4

Table S3: Primer pairs and annealing temperatures used during PCR to target *amoA* or 16S rRNA genes (16S) of the specified target groups.

**Forward primer**	**Reverse primer**	**Target gene**	**Target group**	**T_a (°C)**	**Reference**
CA377F	C576R	*amoA*	Clade A comammox	55	7
Camo1F	Camo846R	*amoA*	Clade A comammox	52	8
CB377F	C576R	*amoA*	Clade B comammox	55	7
AmoA-1F	AmoA-2R	*amoA*	AOB	55	9
Arch-amoAF	Arch-amoAR	*amoA*	AOA	52	10
616F	Univ1492	16S	Bacteria	55	11,12
616F	Ntspa1158R	16S	*Nitrospira*	55	11,13
Arch21F	Univ1492	16S	Archaea	55	14,15

Table S4: 16S rRNA targeted FISH and competitor (comp) probes used in this study. Probe target groups and formamide (FA) concentrations used during hybridization are indicated.

**Probe**	**Sequence (5'-3')**	**Target group**	**FA (%)**	**Reference**
Ntsp0662	GGA ATT CCG CGC TCC TCT	Genus *Nitrospira*	35	16
Ntsp0662 (comp)	GGA ATT CCG CTC TCC TCT		35	16
Ntsp0712	CGC CTT CGC CAC CGG CCT TCC	Phylum *Nitrospirota*	35	16
Ntsp0712 (comp)	CGC CTT CGC CAC CGG TGT TCC		35	16
Nm-OL-703	GCC ATC GAT GTT CTT CCA TAT CTC	*Nitrosomonas* 6a/6b	35	17
EUB338	GCT GCC TCC CGT AGG AGT	Most bacteria	35	18
EUB338-II	GCA GCC ACC CGT AGG TGT	*Planctomycetota*	35	19
EUB338-III	GCT GCC ACC CGT AGG TGT	*Verrucomicrobiales*	35	19

Table S5: Number of active and inactive wells obtained by sorting fluorescently labelled cells from different types of biomass.

Biomass	Description	Positive events (%)	Medium	# wells with sorted cells		
				Inactive	NO_2^- producing	NO_3^- producing
**Bioreactor 1**	Nitrifying tandem reactor, compartment 1	8.5	AOB medium	1	82	1
			NOB medium	44	23	17
**Bioreactor 2**	Nitrifying tandem reactor, compartment 2	19.1	AOB medium	83	1	0
			NOB medium	65	0	19
**Bioreactor 3**	Membrane bioreactor, *Ca.* Nitrospira kreftii enrichment	6.5	AOB medium	84	0	0
			NOB medium	66	0	18
**Bioreactor 4**	Nitrifying membrane bioreactor	32.7	Synt. tap water	74	0	10
**Bioreactor 5**	Anoxic sequencing batch bioreactor	16.6	Synt. groundwater	83	0	1
**Biofilter**	Anaerobic compartment of a RAS biofilter	2.3	Synt. tap water	81	1	2
**Soil**	Soil collected from an agricultural field	4.3	Synt. groundwater	82	0	2
			Synt. tap water	82	1	1

References

1. Koops HP, Böttcher B, Möller UC, Pommerening-Röser A, Stehr G. Classification of eigth new species of ammonia-oxidizing bacteria: *Nitrosomonas communis* sp. nov., *Nitrosomonas ureae* sp. nov., *Nitrosomonas aestuarii* sp. nov., *Nitrosomonas marina* sp. nov., *Nitrosomonas nitrosa* sp. nov., *Nitrosomonas eutropha* sp. nov., *Nitrosomonas oligotropha* sp. nov. and *Nitrosomonas halophila* sp. nov.. Microbiol. 1991; 137:1689–1699.
2. Spieck E, Lipski A. cultivation, growth physiology and chemotaxonomy of nitrite-oxidzing bacteria. In: Klotz MG (ed). Methods in enzymology 2011. Pp 109–130.
3. Flik G, van Rijs JH, Wendelaar Bonga SE. Evidence for high-affinity Ca^{2+}-ATPase activity and ATP-driven Ca^{2+}-transport in membrane Preparations of the Gill Epithelium of the Cichlid Fish *Oreochromis* m*ossambicus*. J. Exp. Biol. 1985; 119:335-347.
4. Poghosyan L, Koch H, Sarkis L, Frank J, van Kessel MAHJ, Jetten MSM, Lücker S. Enrichment of clade B comammox *Nitrispira* using lab-scale trickling filters. Unpublished.
5. van Kessel MAHJ, Speth DR, Albertsen M, Nielsen PH, Op den Camp HJM, Kartal B, et al. Complete nitrification by a single microorganism. Nature 2015; 528:555–9.
6. Mundinger AB, Lawson CE, Jetten MSM, Koch H, Lücker S. Cultivation and transcriptional analysis of a canonical *Nitrospira* under stable growth conditions. Front Microbiol 2019; 10.
7. Jiang R, Wang J-G, Zhu T, Zou B, Wang D-Q, Rhee S-K, An D, J Z-Y, Quan Z-X. Use of newly designed primers for the quantification of comple ammonia-oxidizing (comammox) bacterial clades and strict nitrite oxidizers in the genus *Nitrospira.* Appl Environ Microbiol 2020; 86:e01775-20.
8. Fujitani H, Nomachi M, Takahashi Y, Hasebe Y, Eguchi M, Tsuneda S. Succesfull enrichment of low-abundant comammox *Nitrospira* from nitryfying granules under ammonia-limited conditions. FEMS Microbiol Letters 2020; 367.
9. Rotthauwe JH, Witzel KP, Liesack W. The ammonia monooxygenase structural gene *amoA* as a functional marker: Molecular fine-scale analysis of natural ammonia-oxidizing populations. Appl Environ Microbiol 1997; 63: 4704–4712.
10. Francis CA, Roberts RJ, Beman JM, Santoro AE, Oakley BB. Ubiquity and diversity of ammonia-oxidizingarchaea in water columns and sediments of the ocean. Proc Natl Acad Sci 2005; 102:14683–14688.
11. Juretschko S, Timmermann G, Schmid M, Schleifer K-H, Pommerening-Röser A, Koops H-P et al. Combined molecular and conventional analyses of nitrifying bacterium diversity in activated sludge: *Nitrosococcus mobilis* and *Nitrospira*-like bacteria as dominant populations. Appl Environ Microbiol 1998; 64:3042–3051.

12. Weisburg WG, Barns SM, Pelletier DA, Lane DJ. 16S ribosomal DNA amplification for phylogenetic study. J Bacteriol 1991; 173: 697–703.
13. Maixner F, Noguera DR, Anneser B, Stoecker K, Wegl G, Wagner M et al. Nitrite concentration influences the population structure of *Nitrospira*-like bacteria. Environ Microbiol 2006; 8:1487–1495.
14. Baker GC, Smith JJ, Cowan DA. Review and re-analysis of domain-specific 16S primers. J Microbiol Methods 2003; 55:541–555.
15. Weisburg WG, Barns S, Pelletier DA, Lane DJ. 16S ribosomal DNA amplification for phylogenetic study. J Bacteriol 1991; 173:697–703.
16. Daims H, Nielsen JL, Nielsen PH, Schleifer K-H, Wagner M. *In situ* characterization of *Nitrospira*-like nitrite-oxidizing bacteria active in wastewater treatment plants. Appl Environ Microbiol 2001; 67:5273-5284.
17. Lukumbuzya M, Kristensen JM, Kitzinger K, Pommerening-Röser A, Nielsen PH, Wagner M et al. A refined set of rRNA-targeted oligonucleotide probes for the *in situ* detection and quantification of ammonia-oxidizing bacteria. Water Research 2020; 186:116372.
18. Amann RI, Bunder BJ, Olson RJ, Chisholm SW, Devereuz R, Stahl DA. Combination of 16S rRNA-targeted oligonucleotide probes with flow cytometry for analyxing mixed microbial populations. Appl Environ Microbiol 1990: 56:1919–1925.
19. Daims H, Brühl A, Amann R, Schleifer K-H, Wagner M. The domain-specific probe EUB338 is insufficient for the detection of all bacteria: development and evaluation of a more comprehensive probe set. Sys Appl Microbiol 1999; 22:434–444.
